# Right coronary artery anomaly

**DOI:** 10.4322/acr.2020.242

**Published:** 2021-04-23

**Authors:** Kleio Fragkouli, Theodore Vougiouklakis

**Affiliations:** 1 University of Ioannina, Department of Forensic Medicine and Toxicology, Ioannina, Greece

**Keywords:** Autopsy, Coronary Vessel Anomalies, Adolescent, Death, Sudden

Anomalous origin of the right coronary artery (RCA) from the ascending aorta (high take-off) is uncommon. The prevalence of this anomaly is reported as 0.006% to 0.17%.^1^ ^,^ ^2^ It is notably discovered incidentally during angiography, MRI, or surgery but rarely at autopsy.^1^ ^,^ ^2^ In adolescents and young adults, obstruction to the blood flow due to this RCA anomaly causes ischemia, angina, syncope, or sudden death. Potential symptoms of the right coronary artery anomaly are typically more pronounced with physical/athletic activity.^3^ During exertion, the aortic root suddenly dilates by increasing systolic blood pressure, and this dilation may press the intramural artery decreasing RCA blood flow. Other possible causes of reduced arterial perfusion include ostial obstruction due to slit-like orifice and compression of RCA between the aorta and the right outflow tract.^3^ ^,^ ^4^ Not only is the distance of the anomalous RCA origin from aortic annulus considered responsible for myocardial ischemia and sudden death, but also the intramural course of the artery along with a slit-like orifice.^5^ ^,^ ^6^ The congenital atresia of the right coronary artery ostium is exceedingly rare. Absence of myocardial scarring along the RCA territories is considered as significant finding for congenital, non-acquired, atresia of the right coronary artery ostium.^7^

**Figure 1:**
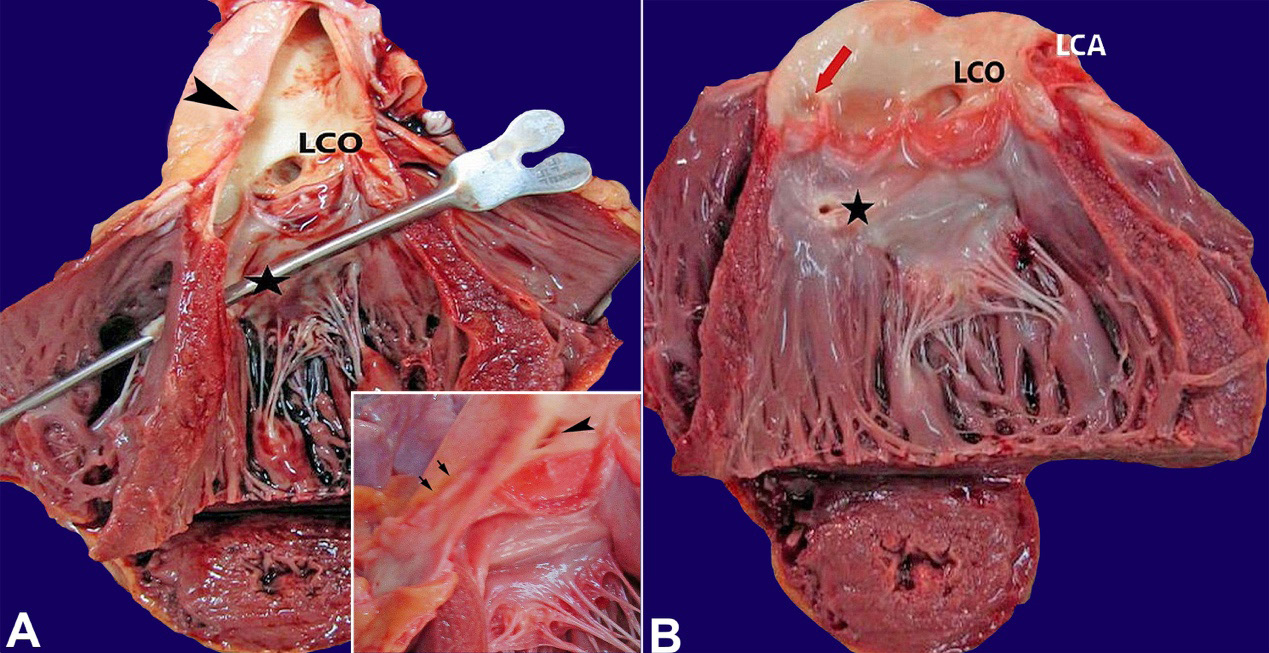
**A**, **B**, and inset - The right coronary artery originated aberrantly from the aortic wall (black arrowhead). The intramural course of the right coronary artery (black arrows in the inset). Congenital atresia of the right coronary artery ostium (red arrow). Defect in the membranous part of the interventricular septum (asterisk). *LCO*: Left coronary ostium. *LCA*: Left coronary artery.

The image above refers to a 16-year-old male, who weighed 55 kg, with no previous medical history, who suddenly collapsed during sports activity. None of his family members had any history of cardiac problems or sudden death. At autopsy, there was no injury on the body. Findings of interest were confined to the heart, which weighed 260 g (mean reference range 276 g for 50 kg). On dissection, congenital atresia of the right coronary ostium was noted. The orifice of the right coronary artery (RCA) was of a slit-like appearance. It was abnormally located on the lateral surface of the aortic wall, on the lesser curvature side of the ascending aorta, approximately 2 cm above the sinotubular junction (Figure 1). The RCA presented a normal lumen and, after its origin, followed an intramural course for a short distance toward the right atrioventricular groove and was discontinued by the postmortem incision. However, most areas of the heart were supplied by branches of the RCA demonstrating the right dominance. In addition, a small defect in the upper interventricular septum, measuring about 3mm in diameter, and a patent foramen ovale were also observed. The left coronary artery normally arose from its ostium, located in the left coronary cusp’s sinus. No myocardial scarring was observed.

We reported a rare case of sudden death due to an infrequent RCA anomaly. In this case, we hypothesize that the dilation of the aorta during exercise combined with the slit-like origin of the RCA led to arterial obstruction and sudden death, through acute myocardial ischemia and malignant ventricular arrhythmia in the RCA territory. Interestingly, the co-existence of the patent foramen ovale and the interventricular septum defect makes the case even rare. The defect in the upper interventricular septum could be either a small muscular ventricular septal defect or a small ventricular septal defect in the lower portion of the membranous septum, which could represent a residual defect associated with spontaneous closure of a previously larger ventricular septal defect. Nonetheless, it is uncertain whether these concurring minor anomalies have played a role in the mechanism of sudden death in this case. To the authors’ knowledge, this is the first case of anomalous origin of the right coronary artery from the aortic wall with atresia of the right coronary ostium accompanied by patent foramen ovale and ventricular septal defect.

Examination of coronary artery anomalies is substantial for anatomical classification of the coronary artery variants. Also, the necessity of recognition of a rare coronary anomaly through diagnostic modalities in order to prevent devastating events in the young is emphasized.
